# 2-Methyl­carbamoyl-4-{4-[3-(trifluoro­meth­yl)benzamido]phen­oxy}pyridinium 4-methyl­benzene­sulfonate monohydrate

**DOI:** 10.1107/S1600536809055603

**Published:** 2010-01-16

**Authors:** Zhao Wang, Na-Na Meng, Ting-Ting Huang, Yong-Kui Zhang, Luo-Ting Yu

**Affiliations:** a State Key Laboratory of Biotherapy and Cancer Center, West China Hospital, West China Medical School, Sichuan University, Chengdu 610041, People’s Republic of China; b Department of Pharmaceutical and Bioengineering, School of Chemical Engineering, Sichuan University, Chengdu 610065, People’s Republic of China

## Abstract

The asymmetric unit of the title compound, C_21_H_17_F_3_N_3_O_3_^+^·C_7_H_7_O_3_S^−^·H_2_O, contains two formula units. In one of the cations, the pyridinium and trifluoro­methyl benzene rings form dihedral angles of 87.42 (8) and 45.92 (8)°, respectively, with the central benzene ring [79.56 (8) and 43.52 (8)° in the other cation]. In the crystal structure, N—H⋯O, O—H⋯O and C—H⋯O hydrogen bonds link the ions and water mol­ecules, forming a three-dimensional network.

## Related literature

For general background to the use of small mol­ecule inhibitors of Raf kinase activity in the treatment of cancer, see: Lowinger *et al.* (2002 ▶). For bond-length data, see: Allen *et al.* (1987 ▶).
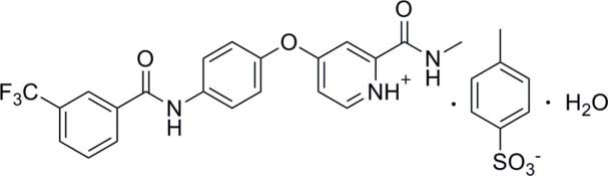


## Experimental

### 

#### Crystal data

C_21_H_17_F_3_N_3_O_3_^+^·C_7_H_7_O_3_S^−^·H_2_O
*M* *_r_* = 605.58
Triclinic, 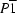

*a* = 10.657 (2) Å
*b* = 16.000 (3) Å
*c* = 16.985 (3) Å
α = 82.98 (3)°
β = 75.63 (3)°
γ = 81.62 (3)°
*V* = 2764.6 (10) Å^3^
*Z* = 4
Mo *K*α radiation
μ = 0.19 mm^−1^
*T* = 113 K
0.29 × 0.25 × 0.22 mm

#### Data collection

Rigaku Saturn CCD area-detector diffractometer
Absorption correction: multi-scan (*ABSCOR*; Higashi, 1995 ▶) *T*_min_ = 0.947, *T*_max_ = 0.960
23339 measured reflections
12940 independent reflections
9042 reflections with *I* > 2σ(*I*)
*R*_int_ = 0.037

#### Refinement

*R*[*F* ^2^ > 2σ(*F* ^2^)] = 0.044
*wR*(*F* ^2^) = 0.112
*S* = 1.02
12940 reflections
802 parameters
H atoms treated by a mixture of independent and constrained refinement
Δρ_max_ = 0.36 e Å^−3^
Δρ_min_ = −0.40 e Å^−3^

### 

Data collection: *CrystalClear* (Rigaku/MSC, 2005 ▶); cell refinement: *CrystalClear*; data reduction: *CrystalClear*; program(s) used to solve structure: *SHELXS97* (Sheldrick, 2008 ▶); program(s) used to refine structure: *SHELXL97* (Sheldrick, 2008 ▶); molecular graphics: *ORTEPIII* (Burnett & Johnson, 1996 ▶); software used to prepare material for publication: *PLATON* (Spek, 2009 ▶).

## Supplementary Material

Crystal structure: contains datablocks global, I. DOI: 10.1107/S1600536809055603/ci2994sup1.cif

Structure factors: contains datablocks I. DOI: 10.1107/S1600536809055603/ci2994Isup2.hkl

Additional supplementary materials:  crystallographic information; 3D view; checkCIF report

## Figures and Tables

**Table 1:** Hydrogen-bond geometry (Å, °)

*D*—H⋯*A*	*D*—H	H⋯*A*	*D*⋯*A*	*D*—H⋯*A*
N1—H1*N*⋯O5^i^	0.90 (2)	2.12 (2)	3.012 (2)	170 (2)
N2—H2*N*⋯O13	0.96 (2)	1.79 (2)	2.664 (2)	150 (2)
N3—H3*N*⋯O12	0.90 (2)	1.90 (2)	2.789 (2)	172 (2)
N4—H4*N*⋯O11^ii^	0.86 (2)	2.06 (2)	2.894 (2)	163 (2)
N5—H5*N*⋯O14	0.93 (2)	1.73 (2)	2.628 (2)	160 (2)
N6—H6*N*⋯O5	0.89 (2)	2.00 (2)	2.868 (2)	163 (2)
O13—H1*O*⋯O9^iii^	0.96 (3)	1.83 (3)	2.785 (2)	173 (2)
O13—H2*O*⋯O10^iv^	0.88 (3)	1.94 (3)	2.802 (2)	167 (2)
O14—H3*O*⋯O4^iii^	1.04 (3)	1.65 (3)	2.682 (2)	172 (2)
O14—H4*O*⋯O3^iii^	0.76 (3)	1.98 (3)	2.737 (2)	179 (3)
C3—H3⋯O8^i^	0.95	2.58	3.477 (2)	158
C4—H4⋯O5^i^	0.95	2.49	3.299 (2)	143
C14—H14⋯O7^v^	0.95	2.48	3.381 (2)	159
C16—H16⋯O12	0.95	2.15	3.068 (2)	162
C18—H18⋯O10^ii^	0.95	2.41	3.220 (2)	143
C18—H18⋯O11^ii^	0.95	2.41	3.061 (2)	126
C24—H24⋯O1^vi^	0.95	2.43	3.300 (2)	151
C38—H38⋯O11^ii^	0.95	2.51	3.293 (2)	139
C44—H44⋯O5	0.95	2.38	3.297 (2)	162
C46—H46⋯O6^ii^	0.95	2.32	2.951 (2)	124
C52—H52⋯O7^vi^	0.95	2.38	3.305 (3)	166

Symmetry codes: (i) 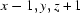; (ii) 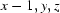; (iii) 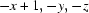; (iv) 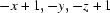; (v) 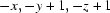; (vi) 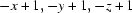.

**Fig. 1.**
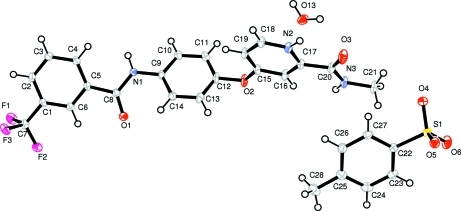
One of the independent formula units of the title compound. Displacement ellipsoids are drawn at the 30% probability level.

## supplementary crystallographic information

### Comment

There are many small molecule inhibitors of Raf kinase activity for the treatment of cancer (Lowinger *et al.*, 2002). The title compound is one of the important agents in our synthetic investigations of antitumor drugs. We report here its crystal structure.

The asymmetric unit of the title compound contains two cationic units, two anionic units and two water molecules. One of the independent units of all constituents are shown in Fig.1. Bond lengths (Allen *et al.*, 1987) and angles are within normal ranges. In one of the cationic units, the central benzene ring (C9-C14) forms dihedral angles of 87.42 (8) and 45.92 (8)°, respectively, with the pyridinium (N2/C15-C19) and trifluoromethyl phenyl ring (C1-C6). The corresponding angles in the other cationic unit are 79.56 (8) and 43.52 (8)°.

In the crystal structure, N—H···O, O—H···O and C—H···O hydrogen bonds link the ionic units and water molecules into a three-dimensional network.

### Experimental

A mixture of 4-methylbenzenesulfonic acid (2.4 g, 14 mmol), ethyl acetate (30 ml) and water (5 ml) was added to a solution of *N*-methyl-4-[4-(3-(trifluoromethyl)benzamido)phenoxy]picolinamide (5 g, 12 mmol) in ethyl acetate (120 ml). The resulting mixture was stirred for 2 h under reflux, then cooled to ambient temperature. The precipitate was collected by filtration and washed with cold ethyl acetate to yield the title compound as a white solid (6.6 g, 91%). Crystals suitable for X-ray analysis were obtained by slow evaporation of a ethyl acetate-water (26:1) solution.

### Refinement

H atoms of the amino group and water molecules were located in a difference map and refined freely. The reminaing H atoms were positioned geometrically (C–H = 0.95–0.98 Å) and refined using a riding model, with *U*_iso_(H) = 1.2–1.5*U*_eq_(C).

### Crystal data

**Table:**

C_21_H_17_F_3_N_3_O_3_^+^·C_7_H_7_O_3_S^−^·H_2_O	*Z* = 4
*M**_r_* = 605.58	*F*(000) = 1256
Triclinic, *P*1	*D*_x_ = 1.455 Mg m^−^^3^
Hall symbol: -P 1	Mo *K*α radiation, λ = 0.71073 Å
*a* = 10.657 (2) Å	Cell parameters from 8045 reflections
*b* = 16.000 (3) Å	θ = 1.7–27.9°
*c* = 16.985 (3) Å	µ = 0.19 mm^−^^1^
α = 82.98 (3)°	*T* = 113 K
β = 75.63 (3)°	Block, colourless
γ = 81.62 (3)°	0.29 × 0.25 × 0.22 mm
*V* = 2764.6 (10) Å^3^	

### Data collection

**Table:**

Rigaku Saturn CCD area-detector diffractometer	12940 independent reflections
Radiation source: rotating anode	9042 reflections with *I* > 2σ(*I*)
confocal	*R*_int_ = 0.037
Detector resolution: 7.31 pixels mm^-1^	θ_max_ = 27.9°, θ_min_ = 1.9°
ω and φ scans	*h* = −13→14
Absorption correction: multi-scan (*ABSCOR*; Higashi, 1995)	*k* = −21→21
*T*_min_ = 0.947, *T*_max_ = 0.960	*l* = −13→22
23339 measured reflections	

### Refinement

**Table:**

Refinement on *F*^2^	Secondary atom site location: difference Fourier map
Least-squares matrix: full	Hydrogen site location: mixed
*R*[*F*^2^ > 2σ(*F*^2^)] = 0.044	H atoms treated by a mixture of independent and constrained refinement
*wR*(*F*^2^) = 0.112	*w* = 1/[σ^2^(*F*_o_^2^) + (0.0554*P*)^2^] where *P* = (*F*_o_^2^ + 2*F*_c_^2^)/3
*S* = 1.02	(Δ/σ)_max_ = 0.001
12940 reflections	Δρ_max_ = 0.36 e Å^−^^3^
802 parameters	Δρ_min_ = −0.39 e Å^−^^3^
0 restraints	Extinction correction: (*SHELXL97*; Sheldrick, 2008), Fc^*^=kFc[1+0.001xFc^2^λ^3^/sin(2θ)]^-1/4^
Primary atom site location: structure-invariant direct methods	Extinction coefficient: 0.0195 (8)

### Special details

**Table:**

Experimental. Interpolation using Int.Tab. Vol. C (1992) p. 523,Tab. 6.3.3.3 for values of muR in the range 0-2.5, and Int.Tab. Vol.II (1959) p.302; Table 5.3.6 B for muR in the range 2.6-10.0. The interpolation procedure of C.W.Dwiggins Jr (Acta Cryst.(1975) A31,146-148) is used with some modification.
Geometry. All e.s.d.'s (except the e.s.d. in the dihedral angle between two l.s. planes) are estimated using the full covariance matrix. The cell e.s.d.'s are taken into account individually in the estimation of e.s.d.'s in distances, angles and torsion angles; correlations between e.s.d.'s in cell parameters are only used when they are defined by crystal symmetry. An approximate (isotropic) treatment of cell e.s.d.'s is used for estimating e.s.d.'s involving l.s. planes.
Refinement. Refinement of *F*^2^ against ALL reflections. The weighted *R*-factor *wR* and goodness of fit *S* are based on *F*^2^, conventional *R*-factors *R* are based on *F*, with *F* set to zero for negative *F*^2^. The threshold expression of *F*^2^ > σ(*F*^2^) is used only for calculating *R*-factors(gt) *etc*. and is not relevant to the choice of reflections for refinement. *R*-factors based on *F*^2^ are statistically about twice as large as those based on *F*, and *R*- factors based on ALL data will be even larger.

### Fractional atomic coordinates and isotropic or equivalent isotropic displacement parameters (Å^2^)

**Table:**

	*x*	*y*	*z*	*U*_iso_*/*U*_eq_	
S1	0.91430 (4)	0.14041 (3)	0.05883 (3)	0.01812 (11)	
F1	−0.34020 (10)	0.61736 (7)	1.15550 (6)	0.0288 (3)	
F2	−0.22027 (10)	0.66570 (6)	1.04205 (7)	0.0285 (3)	
F3	−0.42588 (10)	0.66433 (6)	1.05457 (6)	0.0275 (3)	
O1	0.02637 (12)	0.46121 (8)	0.86161 (8)	0.0272 (3)	
O2	0.40566 (11)	0.19670 (8)	0.63601 (7)	0.0254 (3)	
O3	0.55988 (12)	0.03690 (9)	0.32473 (8)	0.0325 (3)	
O4	0.85481 (12)	0.06433 (7)	0.09516 (8)	0.0260 (3)	
O5	0.87361 (11)	0.17359 (7)	−0.01677 (7)	0.0214 (3)	
O6	1.05425 (11)	0.13110 (8)	0.04777 (8)	0.0285 (3)	
N1	−0.01171 (14)	0.32273 (9)	0.87890 (9)	0.0181 (3)	
H1N	−0.0524 (18)	0.2827 (13)	0.9142 (12)	0.032 (6)*	
N2	0.36243 (13)	0.08092 (10)	0.44574 (9)	0.0200 (3)	
H2N	0.354 (2)	0.0571 (14)	0.3987 (13)	0.045 (7)*	
N3	0.71041 (14)	0.07959 (10)	0.38048 (9)	0.0217 (3)	
H3N	0.7301 (19)	0.1030 (13)	0.4207 (12)	0.032 (6)*	
C1	−0.29467 (16)	0.53268 (10)	1.04459 (10)	0.0167 (3)	
C2	−0.39162 (16)	0.47909 (11)	1.07203 (10)	0.0196 (4)	
H2	−0.4703	0.4974	1.1094	0.023*	
C3	−0.37330 (16)	0.39950 (11)	1.04486 (10)	0.0196 (4)	
H3	−0.4388	0.3626	1.0644	0.024*	
C4	−0.25946 (15)	0.37292 (10)	0.98901 (10)	0.0179 (4)	
H4	−0.2476	0.3182	0.9701	0.022*	
C5	−0.16238 (16)	0.42665 (10)	0.96064 (10)	0.0169 (3)	
C6	−0.18014 (16)	0.50646 (10)	0.98895 (10)	0.0178 (4)	
H6	−0.1141	0.5431	0.9703	0.021*	
C7	−0.31815 (16)	0.61905 (11)	1.07397 (11)	0.0206 (4)	
C8	−0.04016 (16)	0.40543 (11)	0.89624 (10)	0.0186 (4)	
C9	0.09422 (15)	0.29258 (11)	0.81636 (10)	0.0169 (3)	
C10	0.15109 (16)	0.20909 (11)	0.82654 (11)	0.0218 (4)	
H10	0.1198	0.1745	0.8751	0.026*	
C11	0.25314 (17)	0.17617 (11)	0.76617 (11)	0.0242 (4)	
H11	0.2921	0.1192	0.7727	0.029*	
C12	0.29695 (16)	0.22781 (12)	0.69646 (10)	0.0211 (4)	
C13	0.24228 (16)	0.31032 (12)	0.68505 (10)	0.0225 (4)	
H13	0.2744	0.3445	0.6364	0.027*	
C14	0.14031 (16)	0.34301 (11)	0.74490 (10)	0.0199 (4)	
H14	0.1015	0.3999	0.7374	0.024*	
C15	0.38334 (16)	0.15906 (11)	0.57489 (10)	0.0205 (4)	
C16	0.49500 (16)	0.13655 (11)	0.51394 (11)	0.0219 (4)	
H16	0.5783	0.1482	0.5173	0.026*	
C17	0.48196 (16)	0.09764 (11)	0.44978 (10)	0.0194 (4)	
C18	0.25565 (16)	0.10187 (11)	0.50330 (11)	0.0222 (4)	
H18	0.1736	0.0889	0.4989	0.027*	
C19	0.26269 (16)	0.14201 (11)	0.56889 (11)	0.0218 (4)	
H19	0.1862	0.1577	0.6093	0.026*	
C20	0.59060 (17)	0.06918 (11)	0.37853 (10)	0.0214 (4)	
C21	0.82469 (16)	0.05376 (13)	0.31621 (11)	0.0272 (4)	
H21A	0.8044	0.0099	0.2874	0.041*	
H21B	0.8982	0.0312	0.3407	0.041*	
H21C	0.8478	0.1029	0.2776	0.041*	
C22	0.84928 (15)	0.21982 (10)	0.12704 (10)	0.0173 (3)	
C23	0.92194 (17)	0.28484 (11)	0.12897 (11)	0.0226 (4)	
H23	1.0091	0.2839	0.0973	0.027*	
C24	0.86702 (17)	0.35103 (11)	0.17704 (11)	0.0251 (4)	
H24	0.9173	0.3951	0.1781	0.030*	
C25	0.73932 (17)	0.35394 (11)	0.22376 (11)	0.0226 (4)	
C26	0.66884 (17)	0.28718 (11)	0.22277 (11)	0.0229 (4)	
H26	0.5827	0.2871	0.2559	0.027*	
C27	0.72200 (16)	0.22117 (11)	0.17450 (10)	0.0206 (4)	
H27	0.6719	0.1769	0.1737	0.025*	
C28	0.67826 (19)	0.42795 (12)	0.27238 (12)	0.0307 (4)	
H28A	0.7431	0.4670	0.2677	0.046*	
H28B	0.6474	0.4075	0.3298	0.046*	
H28C	0.6045	0.4576	0.2513	0.046*	
S2	0.89838 (4)	0.15086 (3)	0.53643 (3)	0.01848 (11)	
F4	−0.33636 (13)	0.59319 (8)	0.64537 (7)	0.0474 (3)	
F5	−0.20384 (11)	0.64667 (8)	0.54200 (8)	0.0406 (3)	
F6	−0.40474 (11)	0.65049 (8)	0.54127 (8)	0.0467 (3)	
O7	0.01905 (12)	0.47448 (8)	0.32258 (8)	0.0289 (3)	
O8	0.46040 (10)	0.22239 (7)	0.10696 (7)	0.0178 (3)	
O9	0.65314 (11)	0.03829 (8)	−0.18903 (7)	0.0257 (3)	
O10	0.95419 (11)	0.07396 (7)	0.57676 (7)	0.0228 (3)	
O11	0.99564 (11)	0.19081 (8)	0.47245 (7)	0.0251 (3)	
O12	0.78472 (11)	0.13747 (9)	0.50857 (8)	0.0313 (3)	
N4	0.02326 (14)	0.33070 (9)	0.34663 (9)	0.0191 (3)	
H4N	−0.0020 (18)	0.2911 (12)	0.3843 (12)	0.027 (5)*	
N5	0.45081 (13)	0.07662 (9)	−0.07060 (9)	0.0163 (3)	
H5N	0.4452 (19)	0.0439 (13)	−0.1111 (12)	0.035 (6)*	
N6	0.79628 (14)	0.08258 (10)	−0.12880 (9)	0.0205 (3)	
H6N	0.8094 (19)	0.1046 (13)	−0.0860 (12)	0.033 (6)*	
C29	−0.27709 (17)	0.51754 (12)	0.52830 (11)	0.0219 (4)	
C30	−0.36521 (16)	0.45805 (12)	0.55576 (11)	0.0243 (4)	
H30	−0.4409	0.4700	0.5979	0.029*	
C31	−0.34257 (16)	0.38180 (11)	0.52173 (11)	0.0231 (4)	
H31	−0.4028	0.3413	0.5405	0.028*	
C32	−0.23199 (16)	0.36392 (11)	0.46014 (10)	0.0203 (4)	
H32	−0.2167	0.3112	0.4369	0.024*	
C33	−0.14335 (16)	0.42324 (11)	0.43233 (10)	0.0193 (4)	
C34	−0.16670 (16)	0.50029 (11)	0.46667 (10)	0.0203 (4)	
H34	−0.1070	0.5411	0.4479	0.024*	
C35	−0.30389 (18)	0.60065 (12)	0.56420 (11)	0.0265 (4)	
C36	−0.02601 (16)	0.41201 (11)	0.36214 (10)	0.0194 (4)	
C37	0.13333 (15)	0.30592 (10)	0.28415 (10)	0.0168 (3)	
C38	0.19331 (16)	0.22322 (11)	0.29177 (10)	0.0215 (4)	
H38	0.1601	0.1859	0.3378	0.026*	
C39	0.30118 (17)	0.19458 (11)	0.23285 (10)	0.0213 (4)	
H39	0.3430	0.1383	0.2385	0.026*	
C40	0.34650 (15)	0.24888 (10)	0.16636 (10)	0.0163 (3)	
C41	0.28742 (16)	0.33076 (11)	0.15683 (10)	0.0184 (4)	
H41	0.3203	0.3672	0.1100	0.022*	
C42	0.18004 (16)	0.35979 (11)	0.21560 (10)	0.0190 (4)	
H42	0.1385	0.4161	0.2092	0.023*	
C43	0.45035 (15)	0.17280 (10)	0.05117 (10)	0.0142 (3)	
C44	0.56742 (15)	0.14987 (10)	−0.00578 (10)	0.0158 (3)	
H44	0.6470	0.1673	−0.0020	0.019*	
C45	0.56476 (15)	0.10210 (10)	−0.06671 (10)	0.0161 (3)	
C46	0.33890 (16)	0.09730 (10)	−0.01653 (10)	0.0180 (4)	
H46	0.2609	0.0783	−0.0213	0.022*	
C47	0.33494 (15)	0.14550 (10)	0.04570 (10)	0.0173 (3)	
H47	0.2554	0.1599	0.0841	0.021*	
C48	0.67809 (16)	0.07187 (10)	−0.13396 (10)	0.0176 (4)	
C49	0.91145 (16)	0.05350 (13)	−0.19078 (11)	0.0286 (4)	
H49A	0.9047	−0.0039	−0.2026	0.043*	
H49B	0.9900	0.0530	−0.1705	0.043*	
H49C	0.9167	0.0919	−0.2407	0.043*	
C50	0.83936 (16)	0.22327 (11)	0.61150 (10)	0.0187 (4)	
C51	0.91040 (16)	0.28864 (11)	0.61394 (11)	0.0227 (4)	
H51	0.9926	0.2929	0.5768	0.027*	
C52	0.86104 (17)	0.34797 (12)	0.67079 (11)	0.0256 (4)	
H52	0.9097	0.3927	0.6723	0.031*	
C53	0.74038 (18)	0.34208 (12)	0.72563 (11)	0.0254 (4)	
C54	0.67206 (18)	0.27478 (12)	0.72337 (11)	0.0264 (4)	
H54	0.5909	0.2695	0.7614	0.032*	
C55	0.72004 (16)	0.21541 (11)	0.66682 (11)	0.0231 (4)	
H55	0.6722	0.1700	0.6658	0.028*	
C56	0.6846 (2)	0.40946 (13)	0.78350 (12)	0.0356 (5)	
H56A	0.7526	0.4444	0.7842	0.053*	
H56B	0.6525	0.3826	0.8385	0.053*	
H56C	0.6123	0.4453	0.7655	0.053*	
O13	0.25378 (14)	0.01762 (8)	0.34427 (8)	0.0264 (3)	
H1O	0.282 (3)	0.0026 (17)	0.2890 (18)	0.083 (10)*	
H2O	0.182 (2)	−0.0068 (16)	0.3634 (15)	0.064 (8)*	
O14	0.38900 (14)	−0.02701 (9)	−0.15984 (9)	0.0258 (3)	
H3O	0.297 (3)	−0.0464 (16)	−0.1373 (15)	0.072 (8)*	
H4O	0.403 (3)	−0.0305 (17)	−0.2053 (16)	0.061 (9)*	

### Atomic displacement parameters (Å^2^)

**Table:**

	*U*^11^	*U*^22^	*U*^33^	*U*^12^	*U*^13^	*U*^23^
S1	0.0125 (2)	0.0206 (2)	0.0205 (2)	−0.00281 (16)	−0.00165 (16)	−0.00267 (17)
F1	0.0400 (6)	0.0285 (6)	0.0188 (5)	−0.0017 (5)	−0.0061 (5)	−0.0103 (4)
F2	0.0277 (6)	0.0210 (5)	0.0367 (6)	−0.0064 (4)	−0.0023 (5)	−0.0092 (5)
F3	0.0278 (6)	0.0207 (5)	0.0340 (6)	0.0061 (4)	−0.0108 (5)	−0.0059 (5)
O1	0.0274 (7)	0.0193 (6)	0.0304 (7)	−0.0068 (5)	0.0063 (6)	−0.0077 (5)
O2	0.0157 (6)	0.0384 (8)	0.0221 (7)	0.0004 (5)	0.0002 (5)	−0.0160 (6)
O3	0.0228 (7)	0.0542 (9)	0.0224 (7)	−0.0067 (6)	−0.0008 (6)	−0.0172 (6)
O4	0.0254 (7)	0.0191 (6)	0.0299 (7)	−0.0053 (5)	0.0020 (6)	−0.0027 (5)
O5	0.0209 (6)	0.0235 (6)	0.0203 (6)	−0.0028 (5)	−0.0047 (5)	−0.0045 (5)
O6	0.0123 (6)	0.0391 (8)	0.0344 (8)	−0.0004 (5)	−0.0038 (5)	−0.0108 (6)
N1	0.0181 (7)	0.0149 (7)	0.0195 (8)	−0.0028 (6)	0.0003 (6)	−0.0030 (6)
N2	0.0158 (7)	0.0271 (8)	0.0170 (8)	−0.0005 (6)	−0.0034 (6)	−0.0039 (6)
N3	0.0162 (7)	0.0318 (9)	0.0160 (8)	0.0004 (6)	−0.0008 (6)	−0.0082 (7)
C1	0.0190 (9)	0.0158 (8)	0.0158 (8)	0.0017 (7)	−0.0062 (7)	−0.0037 (7)
C2	0.0160 (8)	0.0228 (9)	0.0176 (9)	0.0022 (7)	−0.0017 (7)	−0.0026 (7)
C3	0.0156 (8)	0.0200 (9)	0.0236 (9)	−0.0031 (7)	−0.0045 (7)	−0.0019 (7)
C4	0.0179 (8)	0.0157 (8)	0.0205 (9)	−0.0002 (7)	−0.0050 (7)	−0.0041 (7)
C5	0.0171 (8)	0.0180 (8)	0.0150 (8)	0.0015 (7)	−0.0042 (7)	−0.0028 (7)
C6	0.0181 (8)	0.0184 (8)	0.0172 (8)	−0.0022 (7)	−0.0050 (7)	−0.0007 (7)
C7	0.0205 (9)	0.0215 (9)	0.0196 (9)	−0.0003 (7)	−0.0049 (7)	−0.0031 (7)
C8	0.0183 (8)	0.0190 (8)	0.0184 (9)	−0.0020 (7)	−0.0031 (7)	−0.0036 (7)
C9	0.0151 (8)	0.0204 (8)	0.0156 (8)	−0.0018 (7)	−0.0020 (7)	−0.0063 (7)
C10	0.0230 (9)	0.0202 (9)	0.0208 (9)	−0.0019 (7)	−0.0024 (7)	−0.0027 (7)
C11	0.0253 (10)	0.0210 (9)	0.0255 (10)	0.0033 (7)	−0.0053 (8)	−0.0077 (8)
C12	0.0144 (8)	0.0313 (10)	0.0176 (9)	0.0007 (7)	−0.0013 (7)	−0.0107 (7)
C13	0.0208 (9)	0.0296 (10)	0.0162 (9)	−0.0036 (7)	−0.0016 (7)	−0.0034 (7)
C14	0.0190 (9)	0.0192 (9)	0.0211 (9)	−0.0005 (7)	−0.0043 (7)	−0.0030 (7)
C15	0.0214 (9)	0.0226 (9)	0.0166 (9)	0.0023 (7)	−0.0042 (7)	−0.0049 (7)
C16	0.0131 (8)	0.0305 (10)	0.0215 (9)	−0.0003 (7)	−0.0033 (7)	−0.0046 (8)
C17	0.0168 (8)	0.0241 (9)	0.0158 (8)	0.0015 (7)	−0.0028 (7)	−0.0028 (7)
C18	0.0145 (8)	0.0289 (10)	0.0225 (9)	0.0010 (7)	−0.0043 (7)	−0.0047 (8)
C19	0.0143 (8)	0.0281 (10)	0.0204 (9)	0.0024 (7)	−0.0003 (7)	−0.0057 (7)
C20	0.0190 (9)	0.0282 (10)	0.0161 (9)	−0.0006 (7)	−0.0022 (7)	−0.0048 (7)
C21	0.0184 (9)	0.0386 (11)	0.0197 (9)	0.0026 (8)	0.0032 (7)	−0.0068 (8)
C22	0.0161 (8)	0.0190 (8)	0.0174 (8)	−0.0022 (7)	−0.0057 (7)	−0.0007 (7)
C23	0.0189 (9)	0.0263 (9)	0.0238 (9)	−0.0086 (7)	−0.0049 (7)	−0.0002 (8)
C24	0.0299 (10)	0.0242 (9)	0.0266 (10)	−0.0099 (8)	−0.0130 (8)	−0.0020 (8)
C25	0.0291 (10)	0.0208 (9)	0.0191 (9)	−0.0008 (7)	−0.0100 (8)	0.0002 (7)
C26	0.0203 (9)	0.0242 (9)	0.0226 (9)	−0.0017 (7)	−0.0030 (7)	−0.0020 (8)
C27	0.0181 (9)	0.0210 (9)	0.0228 (9)	−0.0054 (7)	−0.0040 (7)	−0.0002 (7)
C28	0.0410 (12)	0.0257 (10)	0.0281 (10)	0.0005 (9)	−0.0136 (9)	−0.0064 (8)
S2	0.0133 (2)	0.0236 (2)	0.0168 (2)	−0.00178 (16)	−0.00035 (16)	−0.00240 (17)
F4	0.0669 (9)	0.0492 (8)	0.0233 (6)	−0.0057 (7)	0.0001 (6)	−0.0166 (6)
F5	0.0324 (6)	0.0391 (7)	0.0499 (8)	−0.0093 (5)	0.0031 (6)	−0.0229 (6)
F6	0.0403 (7)	0.0371 (7)	0.0691 (9)	0.0175 (6)	−0.0280 (7)	−0.0244 (6)
O7	0.0340 (7)	0.0178 (6)	0.0265 (7)	−0.0013 (5)	0.0079 (6)	−0.0025 (5)
O8	0.0152 (6)	0.0194 (6)	0.0179 (6)	−0.0024 (5)	0.0009 (5)	−0.0078 (5)
O9	0.0219 (7)	0.0366 (8)	0.0201 (7)	−0.0083 (5)	0.0001 (5)	−0.0127 (6)
O10	0.0208 (6)	0.0206 (6)	0.0231 (7)	−0.0024 (5)	0.0005 (5)	0.0012 (5)
O11	0.0220 (6)	0.0270 (7)	0.0193 (6)	−0.0007 (5)	0.0043 (5)	0.0035 (5)
O12	0.0171 (6)	0.0523 (9)	0.0273 (7)	−0.0001 (6)	−0.0057 (6)	−0.0185 (6)
N4	0.0205 (8)	0.0179 (7)	0.0151 (7)	−0.0009 (6)	0.0011 (6)	−0.0002 (6)
N5	0.0159 (7)	0.0184 (7)	0.0156 (7)	−0.0031 (6)	−0.0039 (6)	−0.0034 (6)
N6	0.0148 (7)	0.0281 (8)	0.0183 (8)	−0.0019 (6)	−0.0008 (6)	−0.0080 (7)
C29	0.0210 (9)	0.0266 (9)	0.0180 (9)	0.0043 (7)	−0.0073 (7)	−0.0041 (7)
C30	0.0166 (9)	0.0343 (11)	0.0193 (9)	0.0023 (8)	−0.0007 (7)	−0.0055 (8)
C31	0.0153 (9)	0.0283 (10)	0.0249 (10)	−0.0030 (7)	−0.0043 (7)	0.0005 (8)
C32	0.0197 (9)	0.0209 (9)	0.0205 (9)	0.0019 (7)	−0.0076 (7)	−0.0025 (7)
C33	0.0168 (8)	0.0226 (9)	0.0167 (8)	0.0027 (7)	−0.0037 (7)	−0.0010 (7)
C34	0.0201 (9)	0.0220 (9)	0.0171 (9)	0.0004 (7)	−0.0029 (7)	−0.0012 (7)
C35	0.0228 (10)	0.0337 (11)	0.0215 (10)	0.0013 (8)	−0.0023 (8)	−0.0081 (8)
C36	0.0187 (9)	0.0202 (9)	0.0183 (9)	0.0000 (7)	−0.0035 (7)	−0.0033 (7)
C37	0.0156 (8)	0.0197 (8)	0.0147 (8)	−0.0009 (7)	−0.0021 (7)	−0.0039 (7)
C38	0.0238 (9)	0.0196 (9)	0.0175 (9)	−0.0022 (7)	0.0009 (7)	0.0005 (7)
C39	0.0247 (9)	0.0143 (8)	0.0224 (9)	0.0006 (7)	−0.0020 (7)	−0.0025 (7)
C40	0.0124 (8)	0.0219 (9)	0.0145 (8)	−0.0034 (6)	0.0000 (6)	−0.0056 (7)
C41	0.0202 (9)	0.0198 (8)	0.0144 (8)	−0.0048 (7)	−0.0019 (7)	0.0004 (7)
C42	0.0202 (9)	0.0169 (8)	0.0186 (9)	0.0006 (7)	−0.0041 (7)	−0.0006 (7)
C43	0.0155 (8)	0.0116 (7)	0.0146 (8)	−0.0021 (6)	−0.0013 (6)	−0.0021 (6)
C44	0.0127 (8)	0.0164 (8)	0.0186 (8)	−0.0035 (6)	−0.0036 (7)	−0.0005 (7)
C45	0.0144 (8)	0.0176 (8)	0.0154 (8)	−0.0013 (6)	−0.0035 (7)	0.0010 (7)
C46	0.0143 (8)	0.0210 (8)	0.0185 (9)	−0.0036 (7)	−0.0027 (7)	−0.0015 (7)
C47	0.0127 (8)	0.0195 (8)	0.0179 (8)	−0.0010 (6)	−0.0004 (7)	−0.0026 (7)
C48	0.0171 (8)	0.0181 (8)	0.0161 (8)	−0.0017 (7)	−0.0010 (7)	−0.0025 (7)
C49	0.0173 (9)	0.0412 (12)	0.0252 (10)	−0.0013 (8)	0.0029 (8)	−0.0134 (9)
C50	0.0165 (8)	0.0228 (9)	0.0147 (8)	0.0001 (7)	−0.0023 (7)	0.0001 (7)
C51	0.0146 (8)	0.0283 (10)	0.0236 (9)	−0.0034 (7)	−0.0026 (7)	0.0000 (8)
C52	0.0238 (10)	0.0282 (10)	0.0269 (10)	−0.0049 (8)	−0.0087 (8)	−0.0034 (8)
C53	0.0298 (10)	0.0269 (10)	0.0180 (9)	0.0039 (8)	−0.0065 (8)	−0.0028 (8)
C54	0.0243 (10)	0.0294 (10)	0.0213 (10)	−0.0007 (8)	0.0022 (8)	−0.0039 (8)
C55	0.0208 (9)	0.0238 (9)	0.0217 (9)	−0.0039 (7)	0.0004 (7)	−0.0005 (7)
C56	0.0434 (12)	0.0340 (11)	0.0278 (11)	0.0036 (10)	−0.0063 (9)	−0.0107 (9)
O13	0.0305 (8)	0.0296 (7)	0.0201 (7)	−0.0153 (6)	0.0010 (6)	−0.0070 (6)
O14	0.0305 (8)	0.0305 (7)	0.0196 (7)	−0.0139 (6)	−0.0040 (6)	−0.0064 (6)

### Geometric parameters (Å, °)

**Table:**

S1—O6	1.4440 (12)	S2—O11	1.4590 (13)
S1—O4	1.4532 (13)	S2—C50	1.7648 (19)
S1—O5	1.4703 (13)	F4—C35	1.331 (2)
S1—C22	1.7725 (19)	F5—C35	1.335 (2)
F1—C7	1.343 (2)	F6—C35	1.351 (2)
F2—C7	1.337 (2)	O7—C36	1.224 (2)
F3—C7	1.3560 (19)	O8—C43	1.341 (2)
O1—C8	1.222 (2)	O8—C40	1.4206 (19)
O2—C15	1.347 (2)	O9—C48	1.234 (2)
O2—C12	1.418 (2)	N4—C36	1.361 (2)
O3—C20	1.234 (2)	N4—C37	1.417 (2)
N1—C8	1.365 (2)	N4—H4N	0.864 (19)
N1—C9	1.417 (2)	N5—C46	1.338 (2)
N1—H1N	0.903 (19)	N5—C45	1.355 (2)
N2—C18	1.334 (2)	N5—H5N	0.93 (2)
N2—C17	1.359 (2)	N6—C48	1.321 (2)
N2—H2N	0.96 (2)	N6—C49	1.464 (2)
N3—C20	1.320 (2)	N6—H6N	0.89 (2)
N3—C21	1.466 (2)	C29—C34	1.386 (2)
N3—H3N	0.90 (2)	C29—C30	1.393 (3)
C1—C6	1.391 (2)	C29—C35	1.491 (3)
C1—C2	1.393 (2)	C30—C31	1.378 (3)
C1—C7	1.492 (2)	C30—H30	0.95
C2—C3	1.378 (2)	C31—C32	1.389 (2)
C2—H2	0.95	C31—H31	0.95
C3—C4	1.389 (2)	C32—C33	1.395 (2)
C3—H3	0.95	C32—H32	0.95
C4—C5	1.397 (2)	C33—C34	1.391 (2)
C4—H4	0.95	C33—C36	1.505 (2)
C5—C6	1.390 (2)	C34—H34	0.95
C5—C8	1.504 (2)	C37—C38	1.389 (2)
C6—H6	0.95	C37—C42	1.392 (2)
C9—C10	1.393 (2)	C38—C39	1.386 (2)
C9—C14	1.395 (2)	C38—H38	0.95
C10—C11	1.387 (2)	C39—C40	1.370 (2)
C10—H10	0.95	C39—H39	0.95
C11—C12	1.379 (2)	C40—C41	1.377 (2)
C11—H11	0.95	C41—C42	1.383 (2)
C12—C13	1.374 (2)	C41—H41	0.95
C13—C14	1.380 (2)	C42—H42	0.95
C13—H13	0.95	C43—C47	1.391 (2)
C14—H14	0.95	C43—C44	1.408 (2)
C15—C19	1.383 (2)	C44—C45	1.367 (2)
C15—C16	1.404 (2)	C44—H44	0.95
C16—C17	1.364 (2)	C45—C48	1.509 (2)
C16—H16	0.95	C46—C47	1.372 (2)
C17—C20	1.516 (2)	C46—H46	0.95
C18—C19	1.375 (3)	C47—H47	0.95
C18—H18	0.95	C49—H49A	0.98
C19—H19	0.95	C49—H49B	0.98
C21—H21A	0.98	C49—H49C	0.98
C21—H21B	0.98	C50—C51	1.388 (3)
C21—H21C	0.98	C50—C55	1.393 (2)
C22—C23	1.392 (2)	C51—C52	1.392 (3)
C22—C27	1.394 (2)	C51—H51	0.95
C23—C24	1.387 (3)	C52—C53	1.396 (2)
C23—H23	0.95	C52—H52	0.95
C24—C25	1.392 (2)	C53—C54	1.394 (3)
C24—H24	0.95	C53—C56	1.505 (3)
C25—C26	1.396 (3)	C54—C55	1.387 (3)
C25—C28	1.502 (3)	C54—H54	0.95
C26—C27	1.384 (3)	C55—H55	0.95
C26—H26	0.95	C56—H56A	0.98
C27—H27	0.95	C56—H56B	0.98
C28—H28A	0.98	C56—H56C	0.98
C28—H28B	0.98	O13—H1O	0.96 (3)
C28—H28C	0.98	O13—H2O	0.88 (3)
S2—O10	1.4560 (12)	O14—H3O	1.04 (3)
S2—O12	1.4568 (13)	O14—H4O	0.76 (3)
			
O6—S1—O4	114.09 (8)	O10—S2—O11	112.48 (7)
O6—S1—O5	112.25 (8)	O12—S2—O11	112.90 (8)
O4—S1—O5	111.11 (8)	O10—S2—C50	106.43 (8)
O6—S1—C22	106.77 (9)	O12—S2—C50	105.44 (8)
O4—S1—C22	106.97 (8)	O11—S2—C50	106.64 (8)
O5—S1—C22	105.00 (7)	C43—O8—C40	118.78 (13)
C15—O2—C12	118.33 (14)	C36—N4—C37	125.67 (14)
C8—N1—C9	123.99 (14)	C36—N4—H4N	117.1 (13)
C8—N1—H1N	119.0 (13)	C37—N4—H4N	115.5 (12)
C9—N1—H1N	116.0 (12)	C46—N5—C45	121.95 (16)
C18—N2—C17	121.63 (17)	C46—N5—H5N	115.8 (12)
C18—N2—H2N	119.1 (13)	C45—N5—H5N	122.3 (12)
C17—N2—H2N	119.2 (13)	C48—N6—C49	120.78 (16)
C20—N3—C21	122.60 (16)	C48—N6—H6N	121.5 (13)
C20—N3—H3N	123.8 (13)	C49—N6—H6N	117.6 (13)
C21—N3—H3N	113.5 (13)	C34—C29—C30	120.15 (17)
C6—C1—C2	120.13 (16)	C34—C29—C35	120.23 (17)
C6—C1—C7	121.06 (16)	C30—C29—C35	119.60 (16)
C2—C1—C7	118.78 (15)	C31—C30—C29	119.98 (16)
C3—C2—C1	119.97 (15)	C31—C30—H30	120.0
C3—C2—H2	120.0	C29—C30—H30	120.0
C1—C2—H2	120.0	C30—C31—C32	120.23 (17)
C2—C3—C4	120.29 (16)	C30—C31—H31	119.9
C2—C3—H3	119.9	C32—C31—H31	119.9
C4—C3—H3	119.9	C31—C32—C33	120.09 (17)
C3—C4—C5	120.04 (16)	C31—C32—H32	120.0
C3—C4—H4	120.0	C33—C32—H32	120.0
C5—C4—H4	120.0	C34—C33—C32	119.55 (16)
C6—C5—C4	119.63 (15)	C34—C33—C36	116.51 (16)
C6—C5—C8	116.66 (15)	C32—C33—C36	123.80 (16)
C4—C5—C8	123.61 (16)	C29—C34—C33	120.01 (17)
C5—C6—C1	119.93 (16)	C29—C34—H34	120.0
C5—C6—H6	120.0	C33—C34—H34	120.0
C1—C6—H6	120.0	F4—C35—F5	106.54 (17)
F2—C7—F1	107.09 (15)	F4—C35—F6	105.35 (15)
F2—C7—F3	106.08 (13)	F5—C35—F6	105.35 (16)
F1—C7—F3	105.21 (14)	F4—C35—C29	113.27 (16)
F2—C7—C1	113.63 (14)	F5—C35—C29	113.77 (15)
F1—C7—C1	113.00 (14)	F6—C35—C29	111.86 (16)
F3—C7—C1	111.24 (15)	O7—C36—N4	123.94 (16)
O1—C8—N1	123.59 (16)	O7—C36—C33	119.63 (15)
O1—C8—C5	120.05 (15)	N4—C36—C33	116.43 (15)
N1—C8—C5	116.35 (15)	C38—C37—C42	119.61 (15)
C10—C9—C14	119.63 (15)	C38—C37—N4	117.50 (14)
C10—C9—N1	118.25 (15)	C42—C37—N4	122.86 (14)
C14—C9—N1	122.10 (15)	C39—C38—C37	120.61 (15)
C11—C10—C9	120.33 (16)	C39—C38—H38	119.7
C11—C10—H10	119.8	C37—C38—H38	119.7
C9—C10—H10	119.8	C40—C39—C38	118.84 (15)
C12—C11—C10	118.63 (16)	C40—C39—H39	120.6
C12—C11—H11	120.7	C38—C39—H39	120.6
C10—C11—H11	120.7	C39—C40—C41	121.60 (15)
C13—C12—C11	122.05 (16)	C39—C40—O8	119.96 (14)
C13—C12—O2	118.48 (15)	C41—C40—O8	118.38 (14)
C11—C12—O2	119.40 (15)	C40—C41—C42	119.81 (15)
C12—C13—C14	119.37 (16)	C40—C41—H41	120.1
C12—C13—H13	120.3	C42—C41—H41	120.1
C14—C13—H13	120.3	C41—C42—C37	119.50 (15)
C13—C14—C9	119.99 (15)	C41—C42—H42	120.2
C13—C14—H14	120.0	C37—C42—H42	120.2
C9—C14—H14	120.0	O8—C43—C47	124.88 (15)
O2—C15—C19	125.35 (16)	O8—C43—C44	115.22 (15)
O2—C15—C16	114.78 (16)	C47—C43—C44	119.88 (16)
C19—C15—C16	119.87 (17)	C45—C44—C43	118.96 (16)
C17—C16—C15	118.94 (16)	C45—C44—H44	120.5
C17—C16—H16	120.5	C43—C44—H44	120.5
C15—C16—H16	120.5	N5—C45—C44	119.81 (15)
N2—C17—C16	120.01 (16)	N5—C45—C48	112.88 (15)
N2—C17—C20	113.60 (16)	C44—C45—C48	127.31 (16)
C16—C17—C20	126.40 (16)	N5—C46—C47	121.05 (16)
N2—C18—C19	120.92 (17)	N5—C46—H46	119.5
N2—C18—H18	119.5	C47—C46—H46	119.5
C19—C18—H18	119.5	C46—C47—C43	118.33 (15)
C18—C19—C15	118.63 (16)	C46—C47—H47	120.8
C18—C19—H19	120.7	C43—C47—H47	120.8
C15—C19—H19	120.7	O9—C48—N6	125.13 (16)
O3—C20—N3	125.72 (17)	O9—C48—C45	117.39 (15)
O3—C20—C17	117.36 (16)	N6—C48—C45	117.48 (16)
N3—C20—C17	116.90 (16)	N6—C49—H49A	109.5
N3—C21—H21A	109.5	N6—C49—H49B	109.5
N3—C21—H21B	109.5	H49A—C49—H49B	109.5
H21A—C21—H21B	109.5	N6—C49—H49C	109.5
N3—C21—H21C	109.5	H49A—C49—H49C	109.5
H21A—C21—H21C	109.5	H49B—C49—H49C	109.5
H21B—C21—H21C	109.5	C51—C50—C55	120.47 (17)
C23—C22—C27	119.59 (16)	C51—C50—S2	119.94 (13)
C23—C22—S1	119.91 (13)	C55—C50—S2	119.58 (15)
C27—C22—S1	120.33 (14)	C50—C51—C52	119.92 (16)
C24—C23—C22	120.01 (16)	C50—C51—H51	120.0
C24—C23—H23	120.0	C52—C51—H51	120.0
C22—C23—H23	120.0	C51—C52—C53	120.39 (18)
C23—C24—C25	121.05 (18)	C51—C52—H52	119.8
C23—C24—H24	119.5	C53—C52—H52	119.8
C25—C24—H24	119.5	C54—C53—C52	118.73 (17)
C24—C25—C26	118.24 (17)	C54—C53—C56	121.40 (18)
C24—C25—C28	120.76 (18)	C52—C53—C56	119.83 (19)
C26—C25—C28	120.98 (17)	C55—C54—C53	121.39 (17)
C27—C26—C25	121.28 (17)	C55—C54—H54	119.3
C27—C26—H26	119.4	C53—C54—H54	119.3
C25—C26—H26	119.4	C54—C55—C50	119.06 (18)
C26—C27—C22	119.79 (17)	C54—C55—H55	120.5
C26—C27—H27	120.1	C50—C55—H55	120.5
C22—C27—H27	120.1	C53—C56—H56A	109.5
C25—C28—H28A	109.5	C53—C56—H56B	109.5
C25—C28—H28B	109.5	H56A—C56—H56B	109.5
H28A—C28—H28B	109.5	C53—C56—H56C	109.5
C25—C28—H28C	109.5	H56A—C56—H56C	109.5
H28A—C28—H28C	109.5	H56B—C56—H56C	109.5
H28B—C28—H28C	109.5	H1O—O13—H2O	103 (2)
O10—S2—O12	112.30 (8)	H3O—O14—H4O	105 (2)
			
C6—C1—C2—C3	0.9 (2)	C34—C29—C30—C31	0.1 (2)
C7—C1—C2—C3	178.98 (15)	C35—C29—C30—C31	178.64 (16)
C1—C2—C3—C4	−1.2 (2)	C29—C30—C31—C32	0.1 (3)
C2—C3—C4—C5	0.6 (2)	C30—C31—C32—C33	−0.1 (2)
C3—C4—C5—C6	0.4 (2)	C31—C32—C33—C34	−0.1 (2)
C3—C4—C5—C8	−175.89 (15)	C31—C32—C33—C36	−175.63 (15)
C4—C5—C6—C1	−0.6 (2)	C30—C29—C34—C33	−0.3 (2)
C8—C5—C6—C1	175.86 (14)	C35—C29—C34—C33	−178.83 (15)
C2—C1—C6—C5	0.0 (2)	C32—C33—C34—C29	0.3 (2)
C7—C1—C6—C5	−178.03 (15)	C36—C33—C34—C29	176.15 (14)
C6—C1—C7—F2	−0.2 (2)	C34—C29—C35—F4	−132.90 (17)
C2—C1—C7—F2	−178.28 (14)	C30—C29—C35—F4	48.6 (2)
C6—C1—C7—F1	−122.49 (16)	C34—C29—C35—F5	−11.0 (2)
C2—C1—C7—F1	59.4 (2)	C30—C29—C35—F5	170.46 (15)
C6—C1—C7—F3	119.43 (16)	C34—C29—C35—F6	108.22 (18)
C2—C1—C7—F3	−58.7 (2)	C30—C29—C35—F6	−70.3 (2)
C9—N1—C8—O1	−3.2 (3)	C37—N4—C36—O7	0.9 (3)
C9—N1—C8—C5	175.56 (14)	C37—N4—C36—C33	−179.48 (15)
C6—C5—C8—O1	−12.3 (2)	C34—C33—C36—O7	−22.0 (2)
C4—C5—C8—O1	164.08 (16)	C32—C33—C36—O7	153.64 (18)
C6—C5—C8—N1	168.98 (15)	C34—C33—C36—N4	158.29 (15)
C4—C5—C8—N1	−14.7 (2)	C32—C33—C36—N4	−26.0 (2)
C8—N1—C9—C10	150.58 (17)	C36—N4—C37—C38	161.02 (17)
C8—N1—C9—C14	−31.0 (3)	C36—N4—C37—C42	−20.9 (3)
C14—C9—C10—C11	0.1 (3)	C42—C37—C38—C39	1.7 (3)
N1—C9—C10—C11	178.57 (16)	N4—C37—C38—C39	179.82 (16)
C9—C10—C11—C12	0.2 (3)	C37—C38—C39—C40	−0.9 (3)
C10—C11—C12—C13	−0.3 (3)	C38—C39—C40—C41	−0.1 (3)
C10—C11—C12—O2	176.66 (16)	C38—C39—C40—O8	177.14 (16)
C15—O2—C12—C13	−90.9 (2)	C43—O8—C40—C39	79.4 (2)
C15—O2—C12—C11	92.0 (2)	C43—O8—C40—C41	−103.29 (18)
C11—C12—C13—C14	0.0 (3)	C39—C40—C41—C42	0.3 (3)
O2—C12—C13—C14	−177.00 (16)	O8—C40—C41—C42	−176.93 (15)
C12—C13—C14—C9	0.4 (3)	C40—C41—C42—C37	0.4 (3)
C10—C9—C14—C13	−0.4 (3)	C38—C37—C42—C41	−1.4 (3)
N1—C9—C14—C13	−178.83 (16)	N4—C37—C42—C41	−179.45 (16)
C12—O2—C15—C19	−5.2 (3)	C40—O8—C43—C47	1.0 (2)
C12—O2—C15—C16	174.82 (14)	C40—O8—C43—C44	179.58 (12)
O2—C15—C16—C17	179.65 (16)	O8—C43—C44—C45	−177.48 (14)
C19—C15—C16—C17	−0.3 (2)	C47—C43—C44—C45	1.2 (2)
C18—N2—C17—C16	0.2 (3)	C46—N5—C45—C44	0.5 (2)
C18—N2—C17—C20	−179.84 (14)	C46—N5—C45—C48	−179.72 (13)
C15—C16—C17—N2	−0.3 (3)	C43—C44—C45—N5	−1.0 (2)
C15—C16—C17—C20	179.75 (15)	C43—C44—C45—C48	179.21 (13)
C17—N2—C18—C19	0.5 (3)	C45—N5—C46—C47	−0.1 (2)
N2—C18—C19—C15	−1.1 (3)	N5—C46—C47—C43	0.2 (2)
O2—C15—C19—C18	−178.97 (16)	O8—C43—C47—C46	177.74 (14)
C16—C15—C19—C18	1.0 (3)	C44—C43—C47—C46	−0.8 (2)
C21—N3—C20—O3	0.6 (3)	C49—N6—C48—O9	−0.4 (3)
C21—N3—C20—C17	179.10 (15)	C49—N6—C48—C45	178.83 (14)
N2—C17—C20—O3	1.9 (2)	N5—C45—C48—O9	9.0 (2)
C16—C17—C20—O3	−178.14 (17)	C44—C45—C48—O9	−171.26 (16)
N2—C17—C20—N3	−176.72 (15)	N5—C45—C48—N6	−170.30 (14)
C16—C17—C20—N3	3.2 (3)	C44—C45—C48—N6	9.5 (2)
O6—S1—C22—C23	30.07 (15)	O10—S2—C50—C51	102.11 (14)
O4—S1—C22—C23	152.59 (13)	O12—S2—C50—C51	−138.43 (14)
O5—S1—C22—C23	−89.27 (14)	O11—S2—C50—C51	−18.17 (15)
O6—S1—C22—C27	−154.70 (13)	O10—S2—C50—C55	−79.34 (14)
O4—S1—C22—C27	−32.18 (15)	O12—S2—C50—C55	40.13 (15)
O5—S1—C22—C27	85.95 (14)	O11—S2—C50—C55	160.38 (13)
C27—C22—C23—C24	−0.9 (2)	C55—C50—C51—C52	−1.4 (2)
S1—C22—C23—C24	174.39 (13)	S2—C50—C51—C52	177.11 (12)
C22—C23—C24—C25	−0.2 (3)	C50—C51—C52—C53	0.1 (3)
C23—C24—C25—C26	1.7 (2)	C51—C52—C53—C54	1.4 (3)
C23—C24—C25—C28	−176.91 (16)	C51—C52—C53—C56	−176.45 (16)
C24—C25—C26—C27	−2.4 (3)	C52—C53—C54—C55	−1.6 (3)
C28—C25—C26—C27	176.30 (16)	C56—C53—C54—C55	176.19 (16)
C25—C26—C27—C22	1.4 (3)	C53—C54—C55—C50	0.3 (3)
C23—C22—C27—C26	0.3 (2)	C51—C50—C55—C54	1.2 (2)
S1—C22—C27—C26	−174.97 (13)	S2—C50—C55—C54	−177.34 (13)

### Hydrogen-bond geometry (Å, °)

**Table:**

*D*—H···*A*	*D*—H	H···*A*	*D*···*A*	*D*—H···*A*
N1—H1N···O5^i^	0.90 (2)	2.12 (2)	3.012 (2)	170 (2)
N2—H2N···O13	0.96 (2)	1.79 (2)	2.664 (2)	150 (2)
N3—H3N···O12	0.90 (2)	1.90 (2)	2.789 (2)	172 (2)
N4—H4N···O11^ii^	0.86 (2)	2.06 (2)	2.894 (2)	163 (2)
N5—H5N···O14	0.93 (2)	1.73 (2)	2.628 (2)	160 (2)
N6—H6N···O5	0.89 (2)	2.00 (2)	2.868 (2)	163 (2)
O13—H1O···O9^iii^	0.96 (3)	1.83 (3)	2.785 (2)	173 (2)
O13—H2O···O10^iv^	0.88 (3)	1.94 (3)	2.802 (2)	167 (2)
O14—H3O···O4^iii^	1.04 (3)	1.65 (3)	2.682 (2)	172 (2)
O14—H4O···O3^iii^	0.76 (3)	1.98 (3)	2.737 (2)	179 (3)
C3—H3···O8^i^	0.95	2.58	3.477 (2)	158
C4—H4···O5^i^	0.95	2.49	3.299 (2)	143
C14—H14···O7^v^	0.95	2.48	3.381 (2)	159
C16—H16···O12	0.95	2.15	3.068 (2)	162
C18—H18···O10^ii^	0.95	2.41	3.220 (2)	143
C18—H18···O11^ii^	0.95	2.41	3.061 (2)	126
C24—H24···O1^vi^	0.95	2.43	3.300 (2)	151
C38—H38···O11^ii^	0.95	2.51	3.293 (2)	139
C44—H44···O5	0.95	2.38	3.297 (2)	162
C46—H46···O6^ii^	0.95	2.32	2.951 (2)	124
C52—H52···O7^vi^	0.95	2.38	3.305 (3)	166

Symmetry codes: (i) *x*−1, *y*, *z*+1; (ii) *x*−1, *y*, *z*; (iii) −*x*+1, −*y*, −*z*; (iv) −*x*+1, −*y*, −*z*+1; (v) −*x*, −*y*+1, −*z*+1; (vi) −*x*+1, −*y*+1, −*z*+1.
